# Protective Effect of Quercetin Nanoemulsion on 5-Fluorouracil-Induced Oral Mucositis in Mice

**DOI:** 10.1155/2021/5598230

**Published:** 2021-06-09

**Authors:** Mandana Lotfi, Sohrab Kazemi, Anahita Ebrahimpour, Fatemeh Shirafkan, Marzieh Pirzadeh, Mohammad Hosseini, Ali Akbar Moghadamnia

**Affiliations:** ^1^Student Research Committee, Health Research Center, Babol University of Medical Sciences, Babol, Iran; ^2^Cellular and Molecular Biology Research Center, Health Research Center, Babol University of Medical Sciences, Babol, Iran; ^3^Department of Veterinary Pathology, Babol-Branch, Islamic Azad University, Babol, Iran

## Abstract

The target of this study was to evaluate the efficacy, histopathological, oxidative stress, and molecular effects of quercetin (QRC) in mice with oral mucositis induced by 5-fluorouracil (5-FU). Thirty-six albino male mice with oral mucositis induced by 5-FU as a chemotherapeutic agent were used in this study. The animals were randomly divided into 6 groups: control group, mucositis (MUC) group, pretreatment group, posttreatment group, and two last groups including nanoemulsion form of QRC with a dose of 5 mg/kg in both pretreatment and posttreatment. In the present evaluation, fewer oral lesions were observed in the QRC posttreatment groups compared to the pretreatment and nanoemulsion receiving groups. In the SOD assay, the most significant difference was observed in the posttreatment nanogroup (41.073 ± 1.24) and pretreatment nanogroup (43.453 ± 2.60) in comparison to the 5-FU group (30.897 ± 1.93). The results of CAT assay also showed a significant difference in nano-posttreatment (124.60 ± 10.85), posttreatment (135.4 ± 9.82), and nano-pretreatment groups (128.80 ± 7.20) compared to the 5-FU group (55.07 ± 8.91). The expression of inflammatory genes such as Hif-1*α* and Nf*κ*B in this group was lower than in the other groups, although this difference was not significant. It seems that the use of QRC can improve the treatment process of oral mucositis induced by 5-FU.

## 1. Introduction

There are several drugs available for cancer chemotherapy that can be used alone or in combination with other agents to treat a wide range of malignancies. Although chemotherapy is an efficient process to treat many types of cancer, it usually has toxic side effects depending on the type and dose of the drug. Some side effects of chemotherapy are mild and treatable such as nausea and hair loss, while others can cause serious side effects like infection [1]. The basic mechanism of action of anticancer agents is working on cells with a high rate of division. Based on this mechanism of action, other normal cells with a high rate of division, such as the gastrointestinal epithelium, are affected too. 5-Fluorouracil (5-FU) belongs to the category of anticancer and antimetabolite drugs. 5-FU inhibits DNA synthesis during the S phase of the cell cycle by limiting the availability of thymidylate [2]. It is used alone or in combination with other common drugs in the treatment of various cancers, including breast, head and neck, anal, stomach, colorectal, and some skin cancers [3–5]. The mechanism of action of 5-FU is associated with interference with DNA synthase and inhibition of thymidylate synthase. 5-FU, like other anticancer drugs, has many side effects that reduce the patient's quality of life by causing various destructive effects and sometimes interrupt treatment [6–8]. The most common side effects are nausea, diarrhea, vomiting, oral and intestinal mucositis, mouth ulcers, loss of appetite, light sensitivity, metallic taste, neutropenia, and thrombocytopenia [7]. Oral mucositis (OM) is an important and common side effect of chemotherapy with 5-FU treatment. Its incidence is 40% in chemotherapy and almost 100% in combination with chemotherapy and radiotherapy [9–11]. Chemotherapy-induced OM with anticancer drugs may initiate swelling, erythema, or ulcers. It can also involve a wide range of changes, from mild burning sensitization to painful wounds. OM symptoms consist of sleep and eating disorders, communication barriers, and severe pain, which can affect patients' quality of life [12]. Also, these symptoms can even interrupt the course of treatment [13, 14]. These lesions start when the oral mucosa is exposed to chemical agents, causing DNA destruction and cell death. They are commonly caused by the production of reactive oxygen species (ROS) and oxidative stress [15]. Various studies have shown that the origin and extent of these lesions can be controlled by using antioxidants. Antioxidants protect cells from oxidative stress damage by neutralizing the ROS and preventing their formation [16–18].

Preventing or improving OM can increase the patient's quality of life and uninterrupted therapeutic regimen. Currently, the most important methods used to improve OM include cryotherapy, prescribing nonsteroidal anti-inflammatory drugs (NSAIDs), using mouthwash to disinfect the mouth brushing [12, 19] local anesthesia, such as diphenhydramine and using sodium carbonate mixture of promethazine and milk of manganese [20, 21]. These methods do not seem to be effective enough [22]. Anticancer drugs cause OM by two main mechanisms. In addition to directly damaging the mucosa, these drugs also suppress the immune system and predispose to bacterial infections [23]. Anticancer drugs can reduce the secretion and function of mucosa and mucosal cells [24]. On the other hand, using NSAIDs to alleviate adverse reactions of chemotherapeutic agents is problematic and cannot be a good solution to treat and improve mucositis caused by cancer chemotherapy [25].

**Figure 1:**
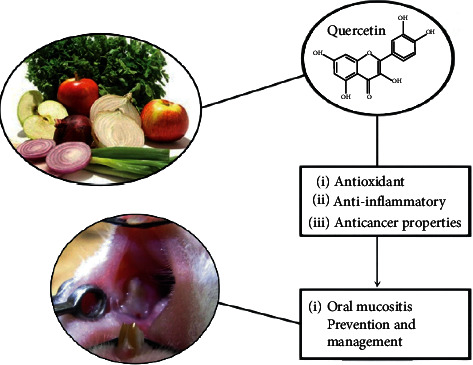
Quercetin is a flavonoid that is abundant in some of our food products that, in addition to treating and preventing cancer, can also be useful in reducing the side effects of chemotherapy such as oral mucositis.

Quercetin (3,3′,4′,5,7-pentahydroxyflavone) (QRC) is a naturally occurring flavonoid found in large quantities in the diet and its main sources are tea, apples, red wine, onions, broccoli, kale, oranges, and blueberries (Figure 1) [26]. The most important property of this flavonoid is its antioxidant effect, but its oral bioavailability is very low. This flavonoid can prevent cell death and oxidative damage by several mechanisms, such as inhibiting the activity of oxygen free radicals, lipid peroxidation, inhibiting xanthene oxidase, and chelating metal ions [27]. It is also known as an antiallergic, anti-inflammatory, and antiviral compound [28]. QRC can prevent the invasion of malignant tumors of the prostate, liver, lung, breast, colon, and cervix. QRC is easily metabolized by the tyrosinase enzyme to different metabolites which can enhance anticancer activity [29]. Recently, it has been reported that QRC in combination with cisplatin, has synergistic effects in cancer treatment [30]. Also, QRC was used in a double-blind, randomized, placebo-controlled clinical trial to prevent and treat OM due to chemotherapy [31]. QRC nanoparticles have been used in many studies to increase the effectiveness of chemotherapy drugs and improve cancer treatment. It has been reported that QRC nanoparticles can reduce injuries caused by intestinal mucositis caused by methotrexate [32]. In another study, QRC nanoparticles were used to improve abnormal cell growth in breast cancer. The results showed that treating with QRC nanoparticles can result in cell death or preventing from cell proliferation in breast cancer [33]. In another study, silica nanoparticles were used to load QRC and doxorubicin nanoparticles, which increased the quality of chemotherapy in gastric cancer [34]. Based on studies and according to the fact that nanoparticles have a large surface area despite their low weight, they can be more active than other molecules; therefore, we used QRC nanoemulsion to improve OM caused by chemotherapy with 5-FU.

## 2. Methods

### 2.1. Drugs and Chemicals

Quercetin (99.9%), glyceryl monooleate (GMO), polyoxyl 40 hydrogenated castor oil, and polyethylene glycol 400 (PEG-400) were purchased from Sigma Aldrich. 5-FU, Tween 80, and saline were prepared from KavoshGostar Daru, Iran.

### 2.2. Nanoemulsion Preparation

A nanoemulsion of QRC formed spontaneously in an oil phase of GMO, PEG-40 hydrogenated castor oil, and PEG 400 (1 : 8 : 1) [35]. 50 mg QRC was added to 10 grams of oil phase. QRC, oil, surfactant, and cosurfactant were stirred at 100 rpm for 2 h. Further sonication for 1 h using a bath sonicator (Elmasonic Med 60) was applied to complete the mixing process. To obtain nanoemulsion, deionized water was added to the oil phase at a ratio of 5 : 1 and stirred gently.

### 2.3. Animal Model and Treatment

Thirty-six adult male albino mice weighing around 25–30 g were accommodated in polyvinyl cages (six mice per cage) at controlled and standard temperature of 22°C ± 3°C with 12-hour light/dark cycle. A standard diet and water were also provided. All experiments and procedures of the study were approved and authorized by the Animal Ethics Committee of the National Institute of Medical Research Development, Tehran, Iran (IR.NIMAD.REC.1398.120). The animals were randomly allocated into six groups:

- Control group (saline + without mucositis)
- Mucositis group (IP injection of a single dose of 300 mg/kg 5-FU in the 6^th^ day) [36]
- Pretreatment group (supplementation with nano-QRC with a dose of 5 mg/kg before the induction of the disease—day 2 to day 6)
- Pretreatment group (supplementation with QRC with a dose of 5 mg/kg before the induction of the disease—day 2 to day 6)
- Posttreatment group (mucositis + supplementation with nano-QRC with a dose of 5 mg/kg after the induction of the mucositis—day 7 to day 13)
- Posttreatment group (mucositis + supplementation with QRC with a dose of 5 mg/kg after the induction of the mucositis—day 7 to day 13)

### 2.4. Drug-Induced Mucositis Model

**Figure 2:**
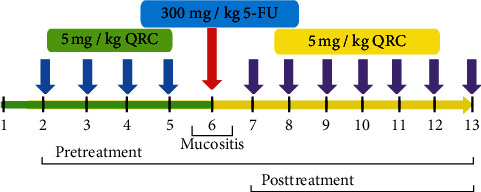
Experimental design of therapeutic efficacy in vivo studies. QRC: quercetin; 5-FU: 5-fluorouracil.

All the experimental mice were kept in the animal house for one day without any treatment to adapt to the environmental conditions. From the second to the sixth day, QRC 5 mg/kg was given intraperitoneally (IP) to pretreatment groups until the 6^th^ day, and on the 6^th^ day, all groups except for the control received a single intraperitoneal (IP) dose 300 mg/kg of 5-FU. After the sixth day of treatment, the pretreatment and posttreatment animals received 5 mg/kg of QRC until the 13^th^ day [37]. During this period, the rate of improvement of the OM of the animals with the scoring method was noted. On the 13^th^ day, the animals were anesthetized with chloroform and their tongues were removed for histological and molecular studies. The severity of inflammation of the oral mucosa and the rate of defecation from the fourth day after induction were assessed by 4 different stages based on previous studies (Figure 2).

### 2.5. Oxidative Stress Measurement

#### 2.5.1. Malondialdehyde (MDA)

Six ml of whole blood from each mice was collected. After clot formation, it was centrifuged at 3000 × g for 10 minutes at 4°C and the serum was separated based on the protocols in the kit. The absorptions of MDA in the plate were read at 530–540 nm by ELISA-reader (TebPazhohanRazi, Tehran, Iran).

#### 2.5.2. Catalase (CAT)

The blood sample was allowed to clot for 10 minutes at 25°C. In order to separate the serum, it was centrifuged at 3000 × g for 10 minutes at 4°C. According to the protocols, the CAT activity was read at 540 nm by ELISA-reader (TebPazhohanRazi, Tehran, Iran).

#### 2.5.3. Superoxide Dismutase (SOD)

The blood sample was allowed to clot for 10 minutes at 25°C. In order to separate the serum, it was centrifuged at 3000 × g for 10 minutes at 4°C. According to the protocols, the SOD activity was read at 440–460 nm wavelength by ELISA-reader (TebPazhohanRazi, Tehran, Iran).

### 2.6. Macroscopic and Microscopic Histopathological Analysis

**Figure 3:**
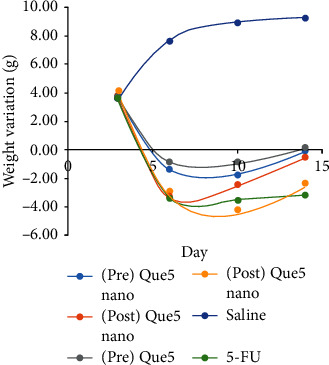
Oral injury score in treatment groups. QRC: quercetin, 5-FU: 5-fluorouracil. The symbols *∗* and *∗∗* indicate *p* < 0.05, 0.01, respectively.

For macroscopic analysis, the changes on the tongue during 4 days were examined. The severity of the injury in a completely blind manner for macroscopic analysis, erythema, vasodilatation, erosion, epithelial ulcerations was evaluated based on a scoring way as follows (Figure 3) [38]:

- Score 0: totally healthy without damage with no erosion or vasodilatation in the surface area.
- Score 1: erythema is present; however, there is no sign of surface erosion
- Score 1.5: existing severe erythema, surface erosion, and presence of vasodilation
- Score 2: focal ulcers in one or more faces of the mucosa are observed, but not exceeding 25% of the surface area, severe erythema and vasodilatation
- Score 2.5: accumulative ulcers can be noticed of about 50% of the surface area
- Score 3: accumulative ulcers can be noticed of about 75% of the surface area

Tissue staining was resolved to consume the hematoxylin and eosin (H&E) experiment. This was accounted for the percentage of H&E-positive cells divided by the total number of cells. Samples were fixed in 10% buffered formalin, fixed in paraffin, and sectioned (in the size of 5 *μ*m). Hematoxylin and eosin were applied for staining.

### 2.7. Real-Time PCR

#### 2.7.1. Sample Collection

The tissue specimens were collected. About 20–30 mg of tongue tissues was transferred immediately to 1.5 ml RNase and DNase-free microtubes including 200 *µ*l RNA later solution (YektaTajhizAzma, Tehran, Iran). After overnight incubation at 4°C, the tissues were transferred to −80°C until the RNA extraction process.

#### 2.7.2. RNA Extraction

Total RNA was extracted from 20 to 30 mg collected tissues using a total RNA extraction kit (Cat No. A101231, Pars Tous Biotechnology, Mashhad, Iran). The isolated RNA samples' amount was measured by a NanoDrop spectrophotometer (Thermo Scientific, Wilmington, USA). All the RNA samples were transferred to −80°C until further experimentation.

#### 2.7.3. cDNA Synthesis and Real-Time PCR

Pars Tous cDNA synthesis kit was applied for cDNA synthesis; the mixture was including 250 ng RNA, 5 *µ*l enzyme buffer 2x, 1 *µ*l of reverse transcriptase enzyme, and the mixture reached the 10 *µ*l volume with DEPC treated water. The thermal program was performed using FleXCycler2 by incubating the reaction mixture 10 minutes at 25°C for random hexamer primer annealing, 60 minutes at 47°C for reverse transcriptase reaction, and 5 minutes at 85°C for ending the reaction. In this study, the SYBR Green method was used for real-time PCR assay. The mRNA amplification was performed by ABI step-one plus PCR system (applied biosystem step-one plus PCR, USA) and using AmpliqonRealQ Plus Master Mix Green-high Rox (Ampliqon, Denmark). The specific primers were designed utilizing Oligo7 v 7.60 software and OligoAnalyzer online tool (http://www.idtdna.com); and the sequence of the primers is represented in Table 1. Every 10 *µ*l PCR reaction mixture consisted of 6.25 *µ*l master mix, 0.25 *µ*l of each primer, 2.25 *µ*l RNase free dH2O, and 1 *µ*l cDNA templates. The PCR temperature protocol was started with 95°C for 15 minutes as the first activation temperature then 40 cycles of temperature were performed at 95°C for 15 s, 61, 62, and 63°C for 30 s (according to the appropriate temperatures for each primer), and 72°C for 30 s and then ramped from 60 to 95°C to achieve a melting curve. In this study, GPDH was used as the reference internal control gene.

### 2.8. Statistical Analysis

All data were analyzed via GraphPad Prism v 8.0.2). Results were expressed as the mean ± SEM. The Kruskal–Wallis test was applied to specify whether there were any statistically significant differences between the means of expression of Hif-1*α* and Nf*κ*B among the groups. Investigation of the mean difference among the groups was carried out by Dunn's multiple comparison test.

## 3. Results

### 3.1. Weight Variation

**Figure 4:**
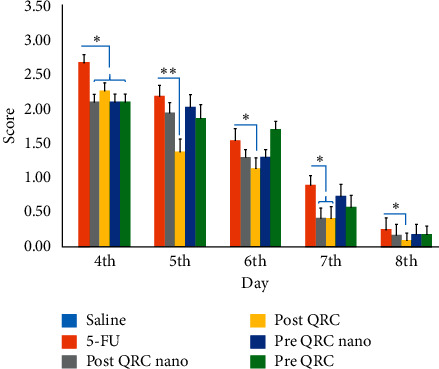
Body weights of mice in the six groups during the experimental period. Que: quercetin; 5-FU: 5-fluorouracil.

During the 13 days of the experiment, analysis of changes in weight over three days (days 6, 10, and 13) showed that the 5-FU group had less weight gain than the control groups (*p* < 0.001, Figure 4). There was also a significant difference between group 5-FU and QRC nano 5 and QRC 5 (*p* < 0.05).

### 3.2. Biochemical Analysis

**Figure 5:**
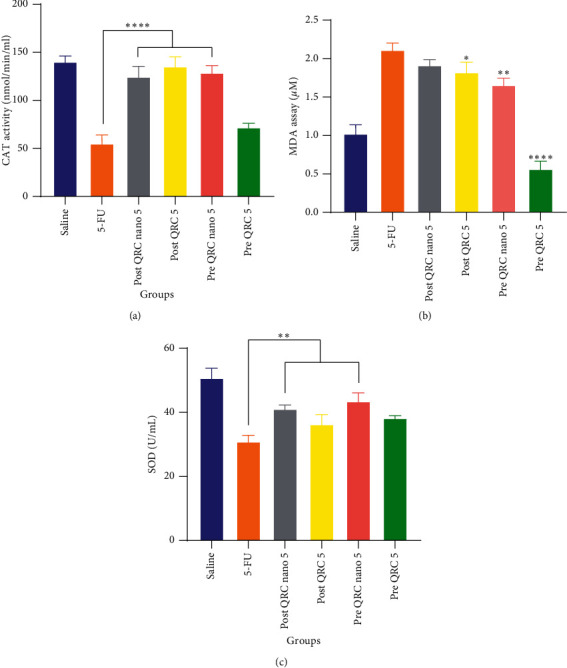
Activity of antioxidant enzymes (a) catalase, (b) malondialdehyde, and (c) superoxide dismutase in serum. The symbol _∗_ indicates the significance of the protective groups compared to the 5-FU group. The symbols ^*∗*^, ^*∗∗*^, ^*∗∗∗*^, and ^*∗∗∗∗*^ indicate *p* < 0.05, 0.01, 0.001, and 0.0001, respectively. CAT: catalase, MDA: malondialdehyde, SOD: superoxide dismutase, QRC: quercetin, and 5-FU: 5-fluorouracil.

The results of the MDA test analysis showed a significant decrease in posttreatment and pretreatment of QRC and nano-pretreatment of QRC in comparison to the 5-FU group (*p* < 0.05, 0.0001, and 0.01, resp.) (Figure 5). In the biochemical analysis of the SOD enzyme, a significant increase was observed between nano-pretreatment and posttreatment of QRC in comparison to the 5-FU group (*p* < 0.01). The results of serum catalase level showed that there was a significant increase between the treatment groups of nano-pretreatment and posttreatment of QRC compared to the 5-FU group (*p* < 0.0001).

### 3.3. Effects of QRC Pretreatment and Posttreatment on Histopathological Aspects of 5-FU-Induced OM

**Figure 6:**
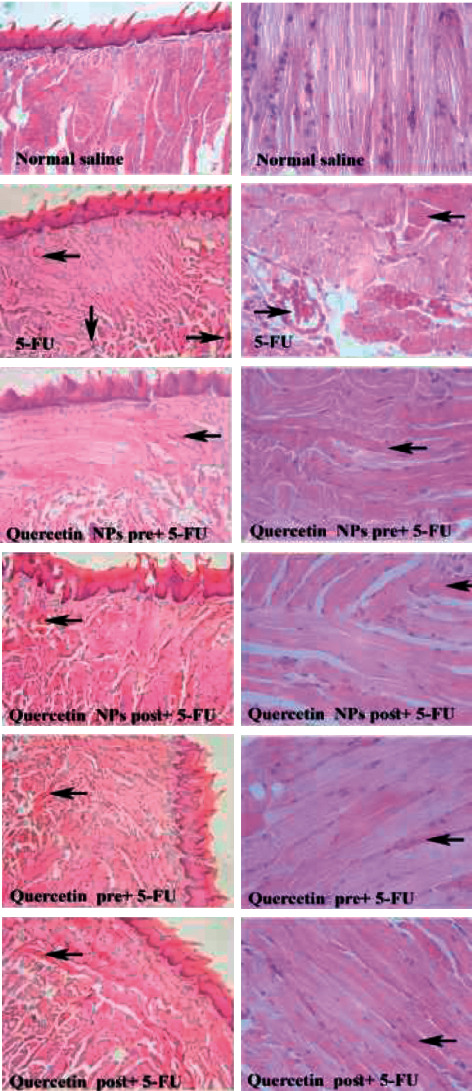
Tongue tissue. Control group: normal tissue conditions, 5-FU group: hyperemia (arrow to the left), hyaline (arrow to the right), infiltration of inflammatory cells (arrow down), other groups: hyalinization (arrow to right), X10, X40 zoom, H&E coloring. QRC: quercetin, 5-FU: 5-fluorouracil, and NPs: nanoemulsion.

The histology of the tongue was normal in the control group, whereas tongue tissue of the 5-FU treatment group had significantly degraded (Table 2) [36]. In the 5-FU group, in addition to hemorrhage, inflammatory cell infiltration and hyalinization occurred in the tongue tissue, although no signs of hemorrhage and infiltration of inflammatory cells were observed in the pretreatment and posttreatment groups (Figure 6).

### 3.4. Evaluation of Hif-1*α* and NF*κ*B Expression in Oral Mucosa Using Real-Time PCR

**Figure 7:**
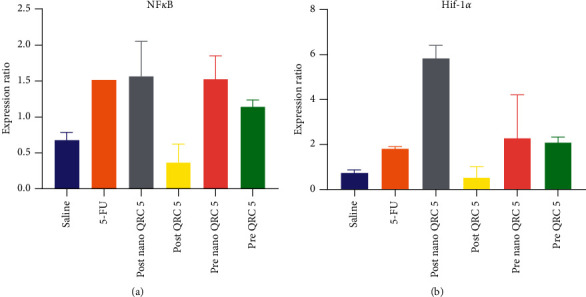
The expression ratio of (a) NF*κ*B and (b) Hif-1*α* in 5-FU injected mice with pre- and post-quercetin nanoemulsion treatment. NF*κ*B: nuclear factor kappa. Hif-1*α*: hypoxia-inducible factor 1-alpha, QRC: quercetin, and 5-FU: 5-fluorouracil.

As the results showed, the expression of Hif-1*α* and NF*κ*B increased in the 5-FU injected group compared to the normal group. The expression of Hif-1*α* and NF*κ*B downregulated in the posttreatment of the QRC group compared to the 5-FU group but not significantly that assign with *p* > 0.9 and *p* > 0.3, respectively. The Hif-1*α* expression in the pretreatment of mice with QRC did not show a considerable difference comparing with the 5-FU group. The downregulation of NF*κ*B was observed in the posttreatment group (Figure 7).

## 4. Discussion

Chemotherapy, which is one of the main therapies of cancer treatment, works by interfering with the synthesis of protein, DNA, and RNA in cells that have a high rate of division [39]. Therefore, normal tissue cells are damaged along with cancer cells. OM is one of the prevalent adverse effects of cancer chemotherapeutic drugs [40]. As mentioned earlier, one of the mechanisms that lead to the complication of OM is the formation of oxidative stress and free radicals. QRC is used in the present study as a powerful antioxidant and anti-inflammatory that has been used against the toxicity of many neoplastic agents [32, 41]. Induction of mucositis is difficult in the animal model, and the extent of the lesion depends entirely on the induction protocol. In this study, we induced OM according to the previous protocol [37]. In mice, the peak severity of oral mucosal lesions usually occurs 4 days after induction and the onset of recovery within 7 days [42]. In the present study, after the administration of 5-FU on day 6, the signs of oral mucositis appeared at around day 10.

Weight loss during the experiment was mostly observed on the fourth day after mucositis, in which QRC pretreatment and QRC pretreatment in nanoform groups showed less weight loss than the 5-FU group, and approximately seven days after mucosal induction, weight gain increased. Weight loss is a common result of treatment with antineoplastic drugs [43].

At the macroscopic level, the results on the 4^th^ day after mucosal induction in all treatment groups showed a significant difference compared to group 5-FU. However, regarding the macroscopic level on the 4^th^ and 6^th^ days after mucosal induction, only the posttreatment of the QRC group showed a significant difference with group 5-FU. However, in histopathology, the posttreatment group with QRC did not show a significant difference compared to the other groups.

CAT and SOD are endogenous antioxidants that are able to clear free radicals. These enzymes' activity and their high levels indicate that the body is under oxidative stress [44]. The performance of the pretreatment groups was more significant in comparison to the posttreatment groups. In both pretreatment groups, a significant decrease in serum MDA level was observed compared to the 5-FU group. Since QRC is widely mentioned in sources as a powerful antioxidant, increasing the strength of the antioxidant system has not been unexpected. Following the results of the present study, some researchers have noted an increase in the number of various antioxidants in other mucosal models [32, 41, 42].

Recent studies have shown that QRC has anti-inflammatory properties and can downregulate the production of some inflammatory factors such as NF*κ*B, COX-2, and NO [44]. In the molecular part of our study, an interesting event occurred. In the QRC posttreatment group, the NF*κ*B level decreased. But in both the nano-pre- and posttreatment of QRC groups, the NF-Κb and Hif-1*α* level increased. To justify this event, we can refer to the synergistic effects of QRC and 5-FU, which we found in a recent study [45]. It means that although the role of certain doses of QRC as a flavonoid with therapeutic properties is undeniable, we can find out that pretreatment with QRC not only cannot prevent its effectiveness against OM damage but also can reduce the levels of antioxidant enzymes and increase the expression of inflammatory factors that lead to cell death and apoptosis.

On the other hand, by comparing the nano- and non-nanoforms of QRC in this study, it can be found that since the nanoform has more permeability to tissues and cells, it was observed that the group receiving 5-FU and nanoform of QRC as the same dose, had poor performance compared to the other groups receiving pre- and posttreatment of QRC. In this case, we found that, to use the nano-QRC form, we had to use a lower dose than the non-nanoform.

Our study focused on comparing the pre- and posttreatment protective effects of QRC against OM caused by 5-FU. QRC, as an effective flavonoid in the treatment of many disorders, if used in inappropriate doses can make disruption on the living body.

## 5. Conclusion

The results of the present study showed that QRC could be a useful compound to prevent the effects of chemotherapy-induced OM with 5-FU, since QRC has been shown to lessen the severity of lesions and inflammation. According to our results from the real-time PCR assay, histopathology, and oxidative stress measurement, using QRC in appropriate doses can be a suitable compound in combating OM.

## Figures and Tables

**Table 1:** The utilized primers sequence.

Hif-1*α* forward	5`- CCCAAGTACCTCAAGAAACGACC- 3`
Hif-1*α* reverse	5`- TGACTCTCTTTCCTGCTCTGTCTG -3`
NF*κ*B forward	5`- AGAGGGGATTTCGATTCCGC -3`
NF*κ*B reverse	5`- CCTGTGGGTAGGATTTCTTGTTC -3`
GAPDH forward	5`- TTGGCATTGTGGAAGGGCTCA -3`
GAPDH reverse	5`- TGGATGCAGGGATGATGTTCTGG -3`

**Table 2:** Results of tissue lesions in different groups.

Groups	Hyperemia	Infiltration of inflammatory cells	Hyaline cells
Ctrl	−	−	−
5-FU	+	+	+
5-FU + quercetin NPs pre	−	−	+
5-FU + quercetin NPs post	−	−	+
5-FU + quercetin pre	−	−	+
5-FU + quercetin post	−	−	+

## Data Availability

Data used to support the study are available from the corresponding author upon request.

## Supplementary Materials

Supplementary Materials A graphical abstract is included in the supporting files.
