# Supplementary material for: Protective Effect of Quercetin Nanoemulsion on 5-Fluorouracil-Induced Oral Mucositis in Mice
Source: J Oncol. 2021 Jun 9;2021:5598230. doi: 10.1155/2021/5598230 (PMC8208858; doi:10.1155/2021/5598230)
Supplement: Supplementary Materials — A graphical abstract is included in the supporting files. [file 5598230.f1.pdf]

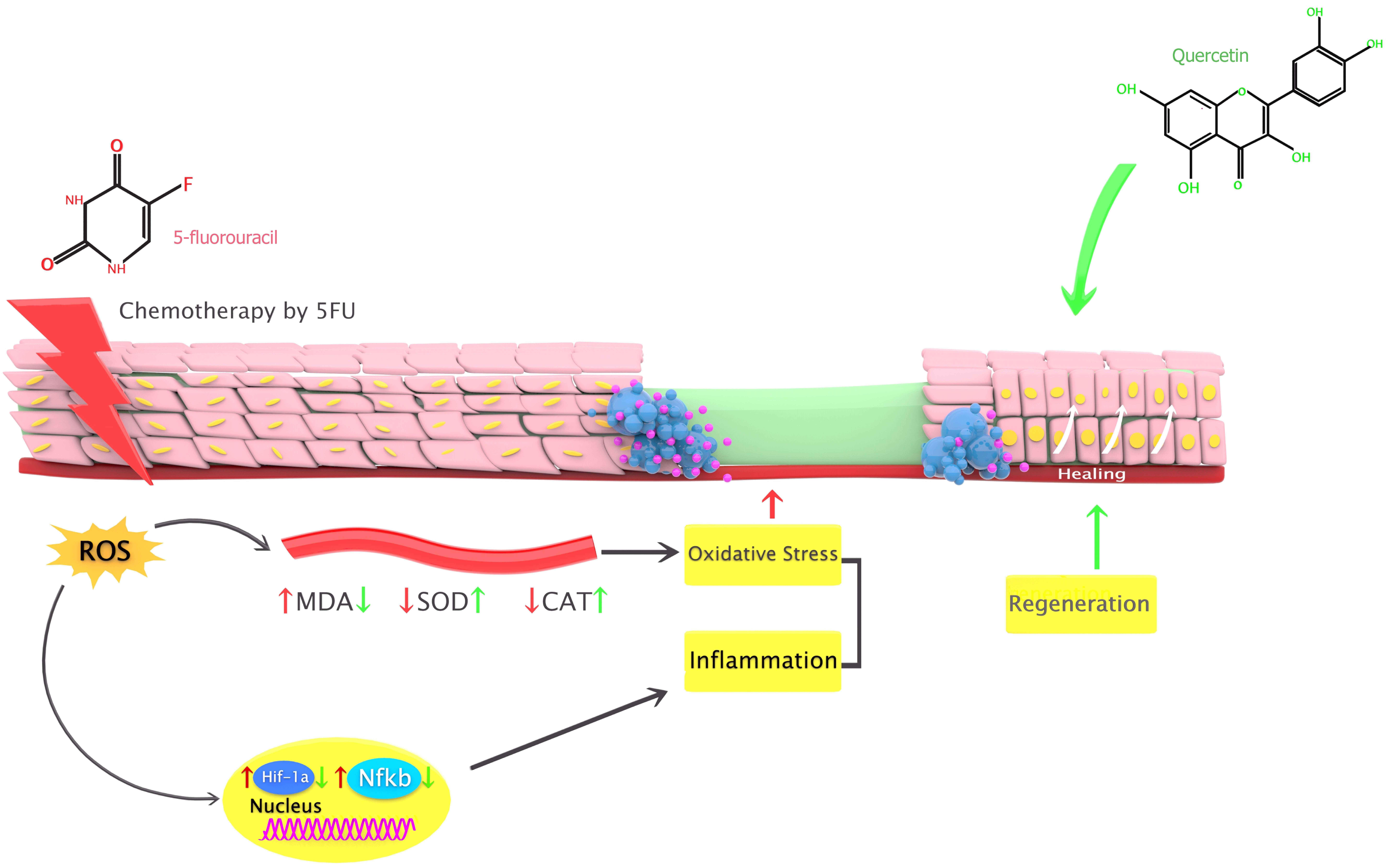

5-fluorouracil
Quercetin
Chemotherapy by 5FU
Healing
ROS
↑MDA↓
↓SOD↑
↓CAT↑
Oxidative Stress
Inflammation
Regeneration
Hif-1a
Nfkb
Nucleus
